# Single-Stage Surgical Treatment of Concurrent Meniere's Disease and Superior Semicircular Canal Dehiscence: A Case Report

**DOI:** 10.7759/cureus.105970

**Published:** 2026-03-27

**Authors:** Nhut Vy P Nguyen, Nicholas Ng, Jeffrey Liaw

**Affiliations:** 1 Otolaryngology, Metropolitan ENT and Facial Plastic Surgery, Alexandria, USA

**Keywords:** endolymphatic sac decompression, meniere’s disease, mixed vertigo, superior semicircular canal dehiscence, vestibular migraine

## Abstract

A 34-year-old woman with concurrent Meniere's disease (MD) and superior semicircular canal dehiscence (SSCD) presented with third-window-mediated drop attacks and episodic vertigo refractory to conservative medical management. The coexistence of these conditions and the pattern of presentation suggested overlapping inner-ear pathophysiology in which abnormal third-window mechanics potentiated MD symptoms. The patient underwent a single-stage transmastoid superior semicircular canal plugging and endolymphatic sac decompression to address both aberrant labyrinthine pressure transmission and impaired endolymphatic regulation. Postoperatively, the patient experienced complete resolution of vertiginous episodes and third-window symptoms without significant impact on hearing. Review of the literature revealed no prior reports of single-stage surgical management of concurrent MD and SSCD. This case describes the complex decision-making and considerations involved in treating a patient with multiple vestibular disorders and highlights the potential role of addressing abnormal intracranial and inner ear pressure dynamics when multiple conditions coexist.

## Introduction

Superior semicircular canal dehiscence (SSCD) and Meniere's disease (MD) are inner-ear disorders affecting both hearing and balance. SSCD is caused by a thinning of the bone overlying the superior semicircular canal, which normally separates it from the intracranial cavity. Cadaveric studies on temporal bones have shown that 0.7% of the general population have a complete dehiscence, and 1.3% have a thin bony layer overlying the superior semicircular canal [1]. In the inner ear, the oval and round windows function as the primary entry and exit points for sound- and pressure-wave transduction. The presence of a defect creates an abnormal “third window,” disrupting this physiologic mechanism, attenuating sound energy transmission, and resulting in a conductive hearing loss [2]. Additionally, aberrant pressure transmission between the third window and the semicircular canal may provoke vertigo. Exposure to loud sounds can also elicit vertigo, known as the Tullio phenomenon [3].

Meniere's disease (MD) is an inner ear disorder characterized by a triad of symptoms, including episodic vertigo, tinnitus, and typically low-frequency sensorineural hearing loss. While the precise etiology of MD remains unknown, it is thought to arise from abnormal accumulation of endolymph within the scala media, causing distention of the membranous labyrinth and disruption of normal cochlear and vestibular signaling, which results in the characteristic auditory and vestibular symptoms. In severe cases of MD, individuals may experience Tumarkin crisis, or drop attacks, caused by sudden pressure changes in the inner ear affecting the utricle and saccule. The diagnosis of MD is clinical and supported by audiometric data. Management of MD focuses on reducing endolymph accumulation, whether through dietary modification (e.g., sodium restriction) or diuretics, with surgery reserved for refractory cases. Surgery is further subdivided into hearing-preserving procedures (endolymphatic sac surgery and vestibular nerve section) and destructive procedures (labyrinthectomy) [4]. 

In this case report, we describe a patient with concurrent ipsilateral MD and SSCD, in whom changes in intracranial pressure and abnormal third-window mechanics were potentiating MD episodes severe enough to present as drop attacks. We describe the single-stage management of both conditions using superior semicircular canal plugging and endolymphatic sac decompression, which resolved vertiginous symptoms with minimal impact on hearing.

## Case presentation

**Figure 1:**
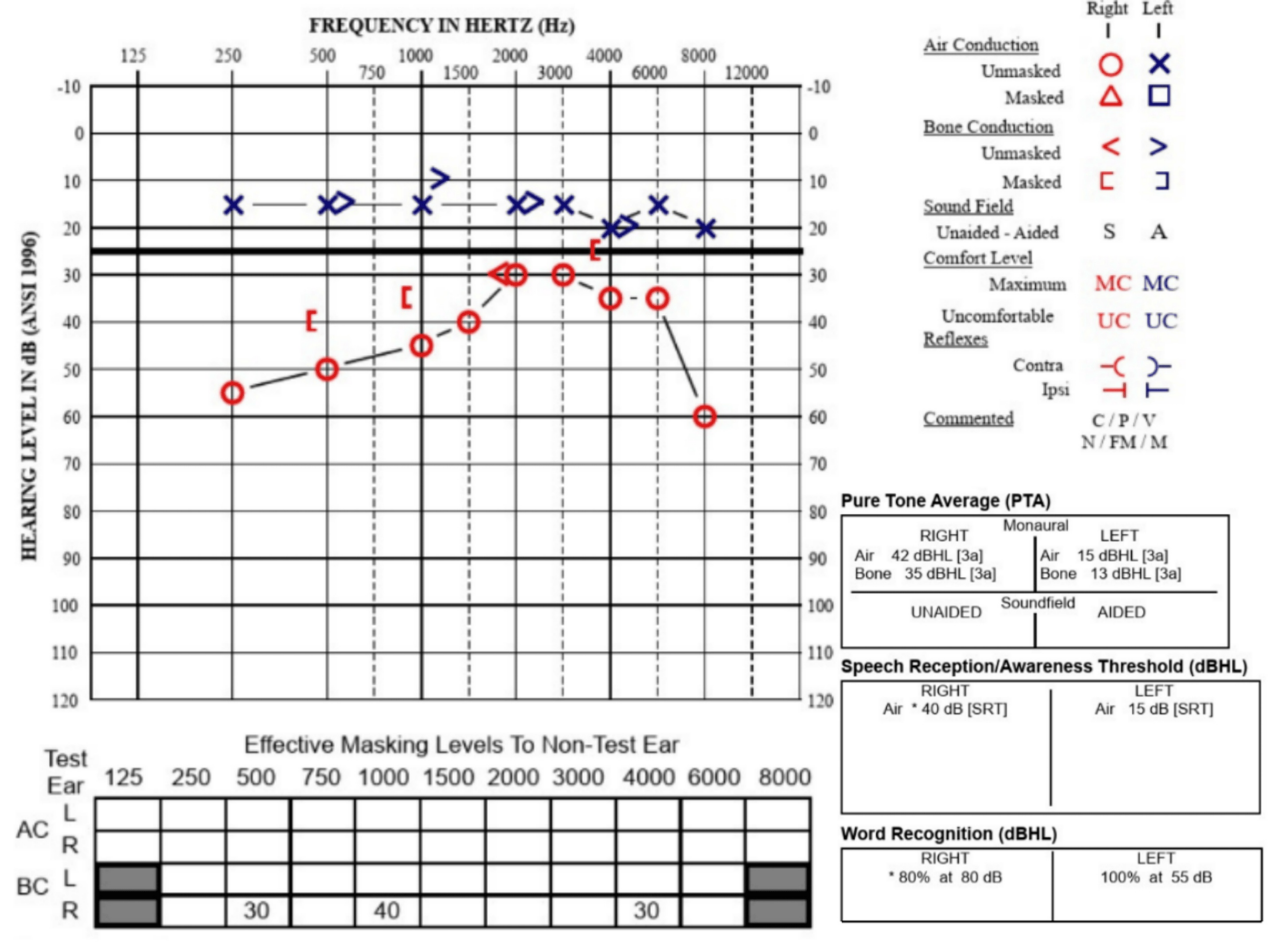
Preoperative audiogram demonstrating a right predominantly low-frequency sensorineural hearing loss.

**Figure 2:**
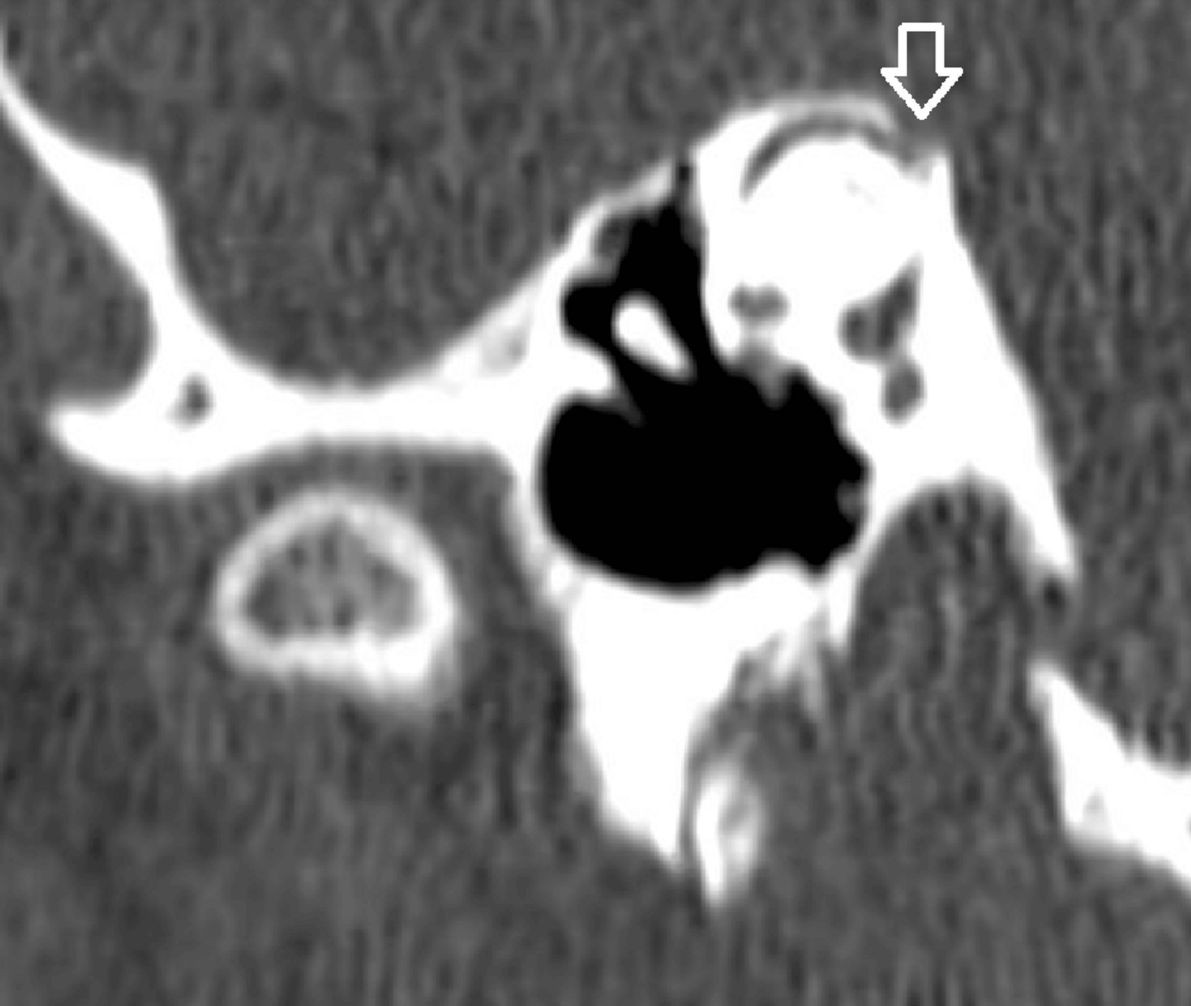
High-resolution CT of the temporal bone in the oblique (Pöschl) plane demonstrating a right superior semicircular canal dehiscence (arrow), measuring approximately 1.5 mm, obtained with a slice thickness of 0.188 mm.

**Figure 3:**
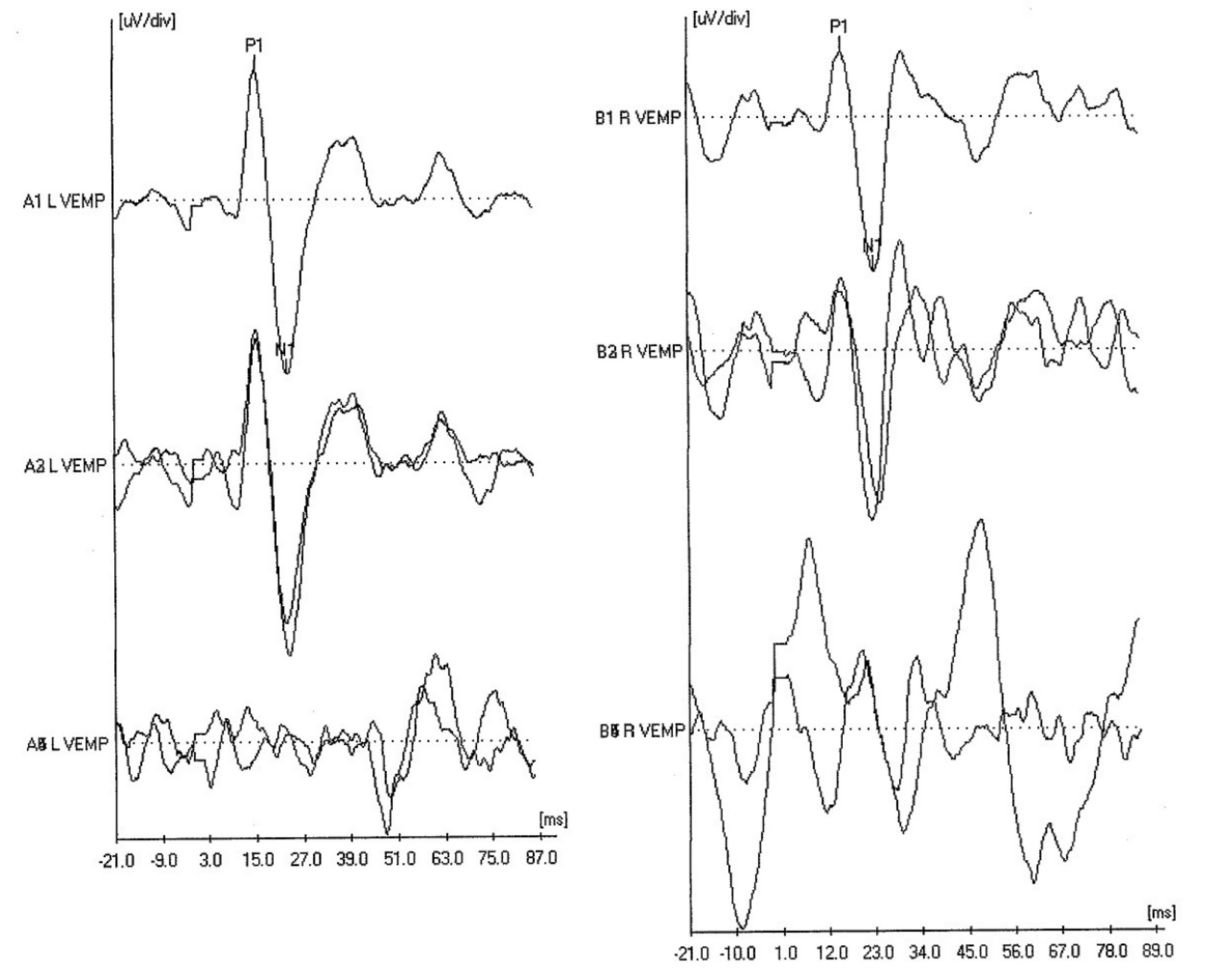
Cervical vestibular evoked myogenic potential demonstrating a response at a low threshold (60 dB) in the right ear, indicating a semicircular canal dehiscence. VEMP: vestibular evoked myogenic potential.

A 34-year-old woman with a history of migraines presented to the clinic with episodic vertigo and low-frequency asymmetrical right sensorineural hearing loss (Figure 1). These vertigo episodes were accompanied by right-sided ear fullness, tinnitus, and fluctuating hearing loss, with symptoms lasting up to half a day. Given these findings, the patient was diagnosed with right-sided MD. An MRI of the internal auditory canal with and without contrast was ordered, which ruled out retrocochlear pathology but raised concern for a right superior semicircular canal dehiscence. The CT temporal bone was subsequently obtained, confirming a right-sided SSCD (Figure 2). Cervical vestibular evoked myogenic potential (cVEMP) obtained demonstrated lower thresholds in the right ear, supporting the finding of right-sided SSCD (Figure 3). Upon further discussion, the patient reported third-window symptoms, in which changes in intracranial pressure (e.g., air travel, heavy lifting, and straining) precipitated immediate vertigo and Ménière’s-associated symptoms, including hearing loss and aural fullness.

To manage symptoms, the patient was started on a Meniere’s diet consisting of low-sodium food and increased hydration. Given the substantial overlap with her migraines, Nortriptyline was started to treat both migraines as well as her Meniere's disease. Additionally, acute attacks were treated with oral steroids. Despite treatment, the patient continued to experience worsening symptoms and developed drop attacks from heavy lifting that resulted in hospitalization.

Due to symptoms refractory to both acute and chronic medical management, as well as her third-window symptoms precipitating MD episodes, the decision was made to proceed with surgical intervention. A transmastoid superior semicircular canal plugging combined with endolymphatic sac decompression was performed to address both her SSCD as well as her MD.

Intraoperatively, the mastoid was exposed through a post-auricular incision and a cortical mastoidectomy was performed using the tegmen, posterior external auditory canal, and sigmoid sinus as the limits of dissection. Incus and lateral canal were identified and kept intact. Donaldson's line, or an imaginary line drawn through the lateral canal and bisecting the posterior canal, was used to identify the endolymphatic sac lying inferior to this line. Bone overlying the sac was removed to visualize the endolymphatic sac as well as for decompression. The tegmen was then skeletonized, and the superior semicircular canal was then identified orthogonal to the lateral canal. Both ascending and descending limbs of the superior semicircular canal were opened and plugged with temporalis fascia and bone wax. The incision was then closed in layers with dissolvable suture.

**Figure 4:**
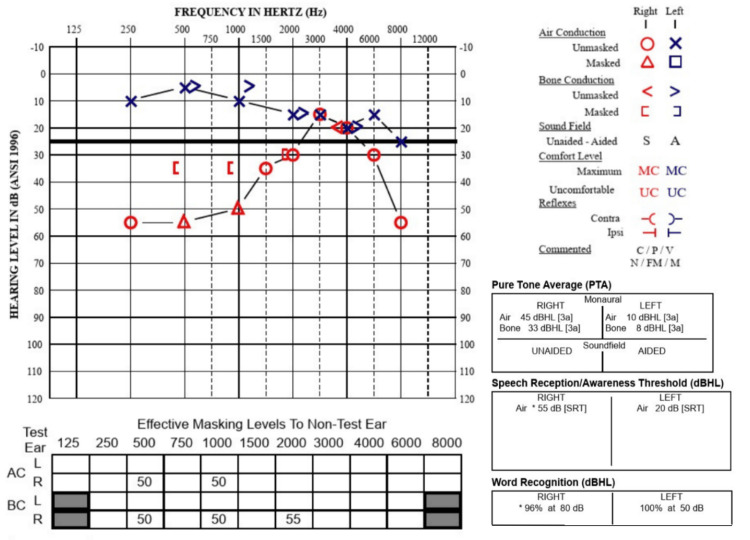
Postoperative audiogram demonstrating stability in right-sided sensorineural hearing loss.

In the immediate postoperative period, the patient reported brief episodes of room-spinning vertigo triggered by head movement, lasting several seconds and relieved by rest. A referral for vestibular therapy was provided; however, all vestibular symptoms self-resolved within three to four weeks, and therapy was ultimately not required. A three-month post-surgical audiogram demonstrated stability in her hearing, with no significant change in hearing noted (Figure 4). At the six-month follow-up, the patient continued to report resolution of the previously described episodic vertigo, right-sided ear fullness, tinnitus, and fluctuating hearing loss symptoms.

## Discussion

This case highlights several key features of MD and SSCD, as well as treatment considerations for patients with multiple vestibular disorders. This patient presented with multiple vestibular symptoms in the setting of worsening migraines. It is known that there is an increased prevalence of migraines in MD, with up to 50% of patients having a history of migraines [5]. Consequently, there have been some authors postulating MD as a manifestation of migraines, theorizing that the recurrent migraine-associated vasoconstriction, vasodilation, and neurogenic inflammation lead to disruptions in inner ear homeostasis, precipitating MD [5,6]. In individuals with concurrent migraines and MD, treatment of MD with migraine prophylactic medications, such as tricyclic antidepressants (nortriptyline) and anticonvulsants (topiramate), has been implemented [4,7]. The patient reported frequent migraines, which increased in frequency since the onset of her MD. Treatment with nortriptyline was therefore initiated to control both her MD attacks as well as her migraines. 

It has been reported that MD can be precipitated by acute changes in barometric pressure [8]. Although the mechanism is not fully elucidated, this is likely due to transmission of pressure from the perilymph into the endolymph. Her SSCD created a third-window effect, amplifying the effects of intracranial forces on her endolymph, resulting in Meniere’s episodes and drop attacks. Successful treatment of her MD was stymied by her SSCD, prompting our decision to proceed with surgical repair. 

Given her vestibular comorbidities, surgical management also presented several challenges. Several techniques for repairing SSCD are described, namely via a middle cranial fossa or transmastoid approach. Additionally, the dehiscence can be covered and resurfaced, or the superior semicircular canal can be plugged to remove input from the canal [9]. In this patient with uncontrolled migraines, we opted to proceed with a transmastoid approach, as a middle cranial fossa approach carries a risk of chronic headaches [10]. In this patient, temporal bone CT demonstrated a bony dehiscence along the posterior-medial (descending) limb of the superior semicircular canal (Figure 2). The distance of the defect relative to the tegmen made a transmastoid resurfacing approach suboptimal. Therefore, a transmastoid superior semicircular canal plugging was planned [9]. 

We also opted for semicircular canal plugging to ablate endolymphatic movement and stabilize labyrinthine fluid dynamics by eliminating the affected canal, thereby removing aberrant vestibular input despite endolymphatic pressure fluctuations. Semicircular canal plugging has been described by multiple authors as an effective means of controlling vertigo in Ménière’s disease despite persistent endolymphatic hydrops [11,12]. The patient was counseled on potential hearing loss and vestibular hypofunction with the possible need for vestibular therapy postoperatively.

As a transmastoid approach was agreed upon, an endolymphatic sac decompression was also performed to further reduce endolymphatic pressure. This procedure is thought to improve fluid homeostasis by alleviating distension of the endolymphatic space, with studies citing a therapeutic success rate between 60% and 90% [13]. Endolymphatic sac decompression was performed concurrently with superior semicircular canal plugging rather than in a staged manner, as the procedure carries relatively low morbidity, is readily performed through the same transmastoid approach, and permits simultaneous treatment of both disease processes to optimize the likelihood of symptom control.

Our review of the literature identified only one prior case series describing patients with concurrent SSCD and MD. However, that study focused primarily on overlapping symptoms of SSCD and MD and did not elaborate on the rationale for management or describe a specific treatment approach [14].

## Conclusions

We report a case of concurrent superior semicircular canal dehiscence and Ménière’s disease presenting with overlapping vestibular symptoms. This case demonstrates that simultaneous superior semicircular canal plugging and endolymphatic sac decompression can be performed safely and effectively as a single-stage surgical approach for vertigo control with minimal morbidity. Importantly, these findings underscore the need to consider combined pathology when disease processes may be mutually reinforcing, and they highlight the potential contribution of intracranial pressure dynamics to the induction or exacerbation of Ménière’s disease.
